# Treatment patterns of individualized real-life tapering approaches based on shared decision-making in rheumatoid arthritis

**DOI:** 10.1007/s00393-023-01380-z

**Published:** 2023-06-23

**Authors:** Benjamin Birkner, Jürgen Rech, Edmund Edelmann, Frank Verheyen, Georg Schett, Tom Stargardt

**Affiliations:** 1 Hamburg Center for Health Economics (HCHE), Universität Hamburg, Esplanade 36, 20354 Hamburg, Germany; 2 Medizinische Klinik 3 – Rheumatologie und Immunologie, Universitätsklinikum Erlangen, Friedrich-Alexander-Universität Erlangen-Nürnberg (FAU), Ulmenweg 18, 91054 Erlangen, Germany; 3 Deutsches Zentrum Immuntherapie, Friedrich-Alexander-Universität Erlangen-Nürnberg and Universitätsklinikum Erlangen, 91054 Erlangen, Germany; 4 Berufsverband Deutscher Rheumatologen e.V, Hauptstraße 9, 13055 Berlin, Germany; 5 Techniker Krankenkasse, Bramfelder Straße 140, 22305 Hamburg, Germany

**Keywords:** Rheumatoid arthritis, Disease-modifying antirheumatic drugs (DMARD’s), Dose tapering, Cohort study, Real world conditions, Rheumatoide Arthritis, krankheitsmodifizierende Antirheumatika (DMARD’s), Dosisreduktion, Kohortenstudie, Reale Bedingungen

## Abstract

**Objective:**

To provide real-world evidence on patient-individual tapering patterns of disease-modifying antirheumatic drugs (DMARDs) in rheumatoid arthritis (RA) patients in daily clinical practice.

**Methods:**

Data obtained through a controlled prospective cohort study in Germany conducted from July 2018 to March 2021 were analyzed. Participants consist of RA patients in sustained remission who were eligible for DMARD tapering at enrolment. Data from RA patients who experienced tapering of DMARDs at least once during the observational period (*n* = 200) were used. Descriptive analyses of medical outcomes at baseline and at time of first tapering, time to first tapering, tapering patterns by substance group, and tapering intensity were documented.

**Results:**

We did not observe meaningful differences in either disease activity or quality of life measures between substance groups at enrolment, time of first tapering, and at 6 or 12 months after tapering. Median time until first tapering varied between substance groups (csDMARDs: 108 days; bDMARDs: 189 days; combination: 119 days). Most patients received one iteration of tapering only (147/200 patients, 73.5%). Dose reduction was applied for patients treated with csDMARDs (79/86 patients, 91.8%), spacing of interval was the most frequent strategy for patients treated with bDMARDs only (43/48 patients, 89.5%). Necessity for increased DMARD dosage was observed in only 10% of patients (20/200). Tapering intensity by substance was overall heterogenous, indicating high individualization.

**Conclusion:**

We identify highly heterogeneous tapering patterns between substance groups and within substances. Identification and recognition of patient-individual approaches of tapering will help to further improve the management of RA for both patients and rheumatologists.

**Supplementary Information:**

The online version of this article (10.1007/s00393-023-01380-z) contains supplementary material, which is available to authorized users.

## Abstract (de)

**Ziel:**

Ziel war es, reale Daten zu patientenbezogenen Ausschleichschemata für krankheitsmodifizierende Antirheumatika (DMARD) bei Patienten mit rheumatoider Arthritis (RA) aus dem klinischen Alltag vorzustellen.

**Methoden:**

Dazu wurden Daten aus einer kontrollierten prospektiven Kohortenstudie in Deutschland untersucht, die von Juli 2018 bis März 2021 durchgeführt wurde. Die Teilnehmer waren RA-Patienten in anhaltender Remission, die sich bei Aufnahme in die Studie als geeignet für ein Ausschleichen der DMARD-Therapie erwiesen. Verwendet wurden die Daten von RA-Patienten, bei denen mindestens einmal während der Beobachtungsphase ein Ausschleichen der DMARD erfolgte (*n* = 200). Deskriptive Analysen der medizinischen Ergebnisse zu Studienbeginn und zum Zeitpunkt des ersten Ausschleichens, die Dauer bis zum ersten Ausschleichen, Ausschleichschemata nach Präparategruppe und Intensität des Ausschleichens wurden dokumentiert.

**Ergebnisse:**

Weder bei der Krankheitsaktivität noch bei den Werten für die Lebensqualität waren bedeutende Unterschiede bei Aufnahme in die Studie, zum Zeitpunkt des ersten Ausschleichens oder 6 bzw. 12 Monate nach dem Ausschleichen zwischen den Präparategruppen festzustellen. Die mittlere Dauer bis zum ersten Ausschleichen variierte zwischen den Präparategruppen: konventionelle synthetische DMARD (csDMARD) 108 Tage, biologische DMARD (bDMARD) 189 Tage, in Kombination 119 Tage. Bei den meisten Patienten erfolgte nur eine einmalige Wiederholung des Ausschleichens (147/200 Patienten, 73,5 %). Während eine Dosisreduktion das Vorgehen bei Patienten unter Therapie mit csDMARD war (79/86 Patienten, 91,8 %), stellte eine Verlängerung des Intervalls die am häufigsten eingesetzte Strategie für Patienten mit alleiniger bDMARD-Therapie dar (43/48 Patienten, 89,5 %). Die Notwendigkeit einer erhöhten DMARD-Dosierung wurde nur bei 10 % der Patienten (20/200) festgestellt. Die Intensität des Ausschleichens nach Präparat war insgesamt heterogen, was ein Zeichen einer hohen Individualisierung ist.

**Schlussfolgerung:**

Im Vergleich zwischen den Präparategruppen und innerhalb der Präparate wurden sehr heterogene Ausschleichschemata beobachtet. Die Identifizierung und das Verständnis patientenindividueller Ansätze beim Ausschleichen von Medikamenten werden dazu beitragen, die Behandlung der RA sowohl für die Patienten als auch für die Rheumatologen weiter zu verbessern.

## Introduction

Clinical remission is the main goal of treatment in patients with rheumatoid arthritis (RA) in clinical practice. International guidelines find broad agreement on therapeutic approaches with disease-modifying antirheumatic drugs (DMARDs) including conventional synthetic (csDMARDs), biologic (bDMARDs), and targeted synthetic substances (tsDMARDs). Central aspects of these guidelines consist of shared decision-making (SDM) between patients and rheumatologists and dose reduction of DMARDs following persistent remission [1–3].

Previous randomized controlled trials (RCTs) have explored possible tapering of DMARDs in RA patients who have achieved sustained remission, revealing the possibility of reducing or withdrawing medication in up to one third of patients [4–7]. However, the application of strictly defined treatment protocols—often halving or withdrawing applied DMARDs—limits the transferability of findings to daily clinical practice.

Using SDM, patients and rheumatologists are able to shape the patient-individual management of RA in mutual agreement. Recent evidence on DMARD tapering following SDM in clinical practice suggests that individual patient characteristics and preferences are drivers of successful tapering [8]. Qualitative research also found that patients prefer rheumatologists offering higher degrees of SDM [9] and rheumatologists tend to increase their efforts regarding SDM when discussing adjustments of drug therapies [10]. In this context, the German guidelines for managing RA highlight the need to explore tapering approaches and the intensity of dose reductions in clinical practice [2].

Using real-world data gathered through a prospective controlled cohort study, we explored tapering patterns outside of clinical studies. This allows us to provide insights into daily clinical practice with respect to tapering DMARDs in Germany. Further, we analyzed whether deviations from fixed reduction regimens, which are often found in RCTs, can be observed for specific substances and/or substance groups. These data inform about the applicability of tapering strategies from RCTs in real-world situations and the potential limitations the treatment in daily practice.

## Methods

### Study setting

We used data from a publicly funded prospective controlled cohort study, *Versorgung von Menschen mit Rheuma optimieren* (VERhO) [11], which measured medical and economic outcomes after patient-individual tapering of DMARDs in a real-world setting in Germany. The recruitment period was July 2018 to September 2020, with a maximum follow-up until the end of March 2021.

Patients were eligible for enrolment if they i) were at least 18 years old, ii) had been treated with any type of DMARDs in the past, iii) had been in stable remission for at least 6 months, and iv) were insured at one of 16 selected health insurance companies that cover about 45% (32.6 million insured) of the German population. Patients receiving glucocorticoids were not eligible for enrolment, except for patients under treatment for adrenocortical insufficiency in accordance with SDM. Further, glucocorticoids were allowed following a flare for short periods with the requirement of successive tapering.

Maximum follow-up is defined from the date of enrolment until the end of the observation period of up to 27 months. Visits were scheduled every 3 months and tapering followed an individual approach as SDM. Spacing of the required 3‑month observation intervals was introduced with the outbreak of COVID-19 in 2020 to limit unnecessary contacts for patients and rheumatologists. A maximum difference of 45 days to the scheduled visits is tolerated for the analyses to account for patient-individual divergence.

The dataset consists of information at the patient visit level covering all prescribed DMARDs with their respective dosage and dosing intervals. Further, we observe disease activity measures consisting of Disease Activity Score 28 based on erythrocyte sedimentation rate (ESR) and C‑reactive protein (CRP) (DAS28-ESR/CRP), Clinical Disease Activity Index (CDAI), patient self-assessed activity based on the Rheumatoid Arthritis Disease Activity Index (RADAI), and quality of life measured by EQ5D-3L.

Following SDM between patients and rheumatologists, written informed consent of patients for participation in VERhO was obtained by rheumatology practices. VERhO was approved by the ethics committee of the University of Erlangen-Nuremberg.

### Statistics

Information of the per-protocol population consisting of patients from the intervention group who initiated tapering within the observation period is used to investigate the application of individual treatment patterns under real-world conditions. We stratify the population by substances and substance groups (csDMARDs, bDMARDs, and tsDMARDs). Individual dose reductions are identified based on the difference between average daily doses in milligrams at subsequent visits per patient.

Identification of tapering patterns is done with alluvial diagrams depicting the flow of patients through different states at 3‑month intervals within the first 12 months after first tapering. We define five states consisting of titration, spacing, mixed, multiple adjustments, no change, and unobserved. *Titration* is defined as any dose reduction of the same substance between two consecutive visits without changes in drug administration intervals. *Spacing* describes longer drug administration intervals while the applied dose is kept constant. *Mixed* denotes cases where both the dosage as well as the administration interval were changed at the same visit resulting in an overall lower dosage. If either *tapering* or *spacing* is identified for more than one substance, the patient is categorized as having received *multiple adjustments *which are only applicable for patients who are treated with combination therapy of DMARDs at baseline. *No change* consists of unchanged administration of DMARDs between two consecutive visits. Missing observations are categorized as *unobserved.*

Population characteristics at enrolment and at first tapering are analyzed with descriptive summary statistics and presented by substance groups. Tapering intensity is presented by the substance.

## Results

### Study population

**Fig. 1:**
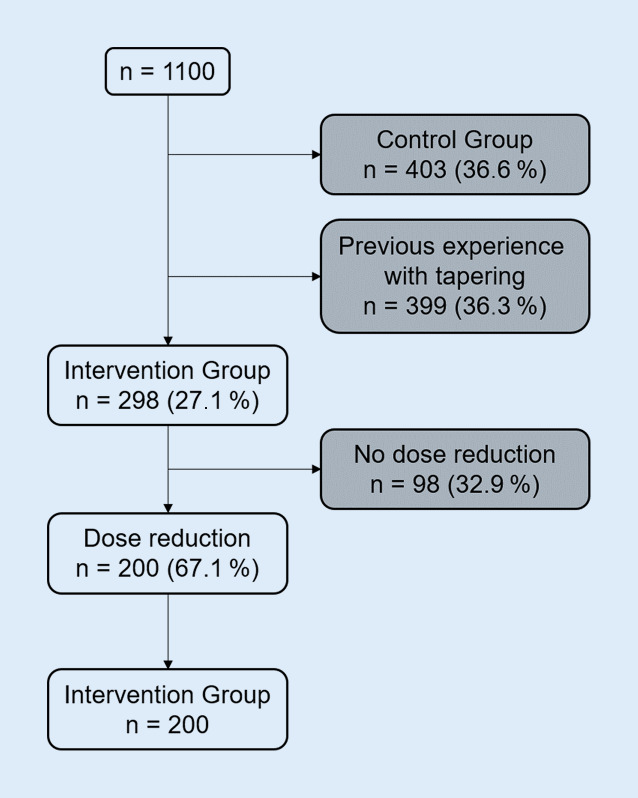
Population flowchart based on the recruited population

Overall, 1100 patients were enrolled by 47 rheumatologists from 24 practices between July 2018 and December 2020. We restrict our analysis to those who were allocated for dose tapering to the intervention group (*n* = 298), whereas those who had tapered DMARDs before (*n* = 399) as well as those allocated to the control group with continuous treatment with DMARDs (*n* = 403) were excluded. The exclusion of patients without any dose reductions despite being allocated to the intervention group (*n* = 98) concludes the selection process. The final study population consists of 200 patients from the intervention group who received first tapering throughout the observation period (Fig. 1).

Patients in monotherapy with either csDMARD or bDMARD constituted the majority of the study sample, with 86/200 patients (43%) and 52/200 patients (26%), respectively. 48/200 patients (24%) received combination therapy of one bDMARD with one csDMARD; tsDMARD monotherapy and combination of one tsDMARD with one csDMARD reflected a minority, with only seven patients (3.5%) each. Mean descriptive statistics at baseline and at time of first tapering are reported in Table 1.

**Table 1:** Descriptive statistics of study population at baseline and at first tapering

	csDMARD *n* = 86		bDMARD *n* = 48		csDMARD and bDMARD *n* = 52		tsDMARD *n* = 7		csDMARD and tsDMARD *n* = 7		Overall *n* = 200	
	Mean (*n*)	sd (%)	Mean (*n*)	sd (%)	Mean (*n*)	sd (%)	Mean (*n*)	sd (%)	Mean (*n*)	sd (%)	Mean (*n*)	sd (%)
*Baseline*												
Age	61.7	12.2	52.1	17.6	54.0	13.8	62.1	6.9	62.0	13.3	57.4	14.5
Female	66	76.7	37	78.7	39	75.0	4	57.1	5	71.4	151	75.9
RA duration	6.0	5.8	12.5	9.2	12.21	10.7	9.78	5.7	5.15	3.1	9.29	8.7
DAS28	2.00	0.66	1.80	0.66	1.93	0.78	2.18	1.03	2.11	0.69	1.96	0.71
DAS28-CRP	1.86	0.47	1.70	0.36	1.78	0.50	2.07	0.65	1.89	0.33	1.81	0.46
CDAI	2.31	2.39	1.93	1.68	2.14	1.92	3.01	3.30	2.36	1.32	2.20	2.12
Remission	81	95.3	47	100.0	46	93.9	6	85.7	7	100.0	187	95.9
Seropositive	22	45.8	19	65.5	19	67.9	4	80.0	2	50.0	66	57.9
EQ5D	0.93	0.12	0.91	0.08	0.93	0.07	0.90	0.04	0.95	0.06	0.93	0.09
RADAI	1.18	1.13	1.47	1.39	1.21	0.96	1.22	0.92	1.01	0.64	1.25	1.14
*First tapering*												
DAS28	2.00	0.63	2.02	0.76	1.95	0.69	2.54	0.50	1.80	0.53	2.00	0.67
DAS28-CRP	1.85	0.45	1.76	0.52	1.75	0.47	1.64	0.70	1.90	0.58	1.79	0.49
CDAI	2.28	2.21	2.66	2.66	2.22	2.72	2.14	2.74	2.53	2.59	2.36	2.47
Remission	80	93.0	45	93.8	50	96.2	6	85.7	6	85.7	187	93.5
EQ5D	0.93	0.09	0.91	0.11	0.91	0.10	0.94	0.08	0.98	0.05	0.92	0.10
RADAI	1.19	1.09	1.43	1.31	1.30	1.28	1.22	1.16	1.05	1.03	1.28	1.19

*csDMARD *conventional synthetic disease-modifying antirheumatic drug, *bDMARD *biological DMARD, *tsDMARD *targeted-synthetic DMARD, *sd* standard deviation

Patients treated with csDMARD monotherapy, tsDMARD only, and csDMARD + tsDMARD were older (61.7, 62, and 62.1 years, respectively) compared to patients under bDMARD monotherapy (52 years) or patients on combination therapy with csDMARD + bDMARD (54 years). Disease activity based on DAS28-ESR, DAS28-CRP, and CDAI was similar between groups, while disease duration was lower in the csDMARD (6 years) and in the csDMARD + tsDMARD group, with 5.15 years as in contrast to the three other groups (12.5 years [bDMARD]; 12.21 years [csDMARD + bDMARD], and 9.78 years [tsDMARD]) The fraction of patients in remission, defined as DAS28 < 2.6 or CDAI < 2.8, also showed no differences between patients in monotherapy with cs/bDMARDs or combination therapy of a csDMARD and a bDMARD. All patients reported a very high quality of life, with values between 0.91 and 0.93 on average. Comparison between the time of enrolment and first tapering did not reveal meaningful differences in disease activity or quality of life, which indicates good disease control prior to the first tapering iteration.

DAS28-ESR, DAS28-CRP, and quality of life remained largely stable for 6 months and 12 months after first tapering. Disease activity based on CDAI, however, appears to increase 12 months after first tapering to values past the remission threshold of 2.8.

Initial tapering following titration or spacing strategies of single substances was applied for the vast majority of patients. 122/200 (61%) patients received titration and 72/200 (36%) spaced administration of DMARDs. Mixed approaches by simultaneously changing dosage and timing were applied for two patients only, while more than one substance was adjusted for three patients overall. A dose increase of the given csDMARD was observed for one patient who simultaneously reduced the bDMARD dose.

The analysis by substance group is restricted to patients within the substance groups of csDMARDs (*n* = 86), bDMARDs (*n* = 48), and the combination of csDMARDs and bDMARDs (*n* = 52), due to the low number of observable patients treated with tsDMARDs (*n* = 14) in our sample.

### Time to first tapering

Time to first tapering varied substantially between substance groups. Median time until first tapering was lowest for patients treated with csDMARDs only, with 108 days (IQR: 111 days). Patients with combination treatments of csDMARDs and bDMARDs showed a longer median time to first tapering (119 days; IQR: 142 days). The largest variation in time until first tapering was found in patients treated with bDMARDs only at first tapering with a median of 189 days (IQR: 287 days). Overall, the observed time to first tapering suggests rather conservative initiation of tapering, since all patients were required to have been in remission for 6 months at baseline, which would have enabled the immediate begin of tapering after enrolment.

### Tapering patterns by substance groups

The type of tapering strategy differed between substance groups. 79/86 patients (91.8%) treated with csDMARDs only received tapering in the form of titration, while spacing was chosen for 43/48 (89.5%) patients treated with bDMARDs. If treated with the combination of one csDMARD and one bDMARD, 22/52 (42.3%) received spaced administration and 26/52 (50%) received titration of one of the two substances.

**Fig. 2:**
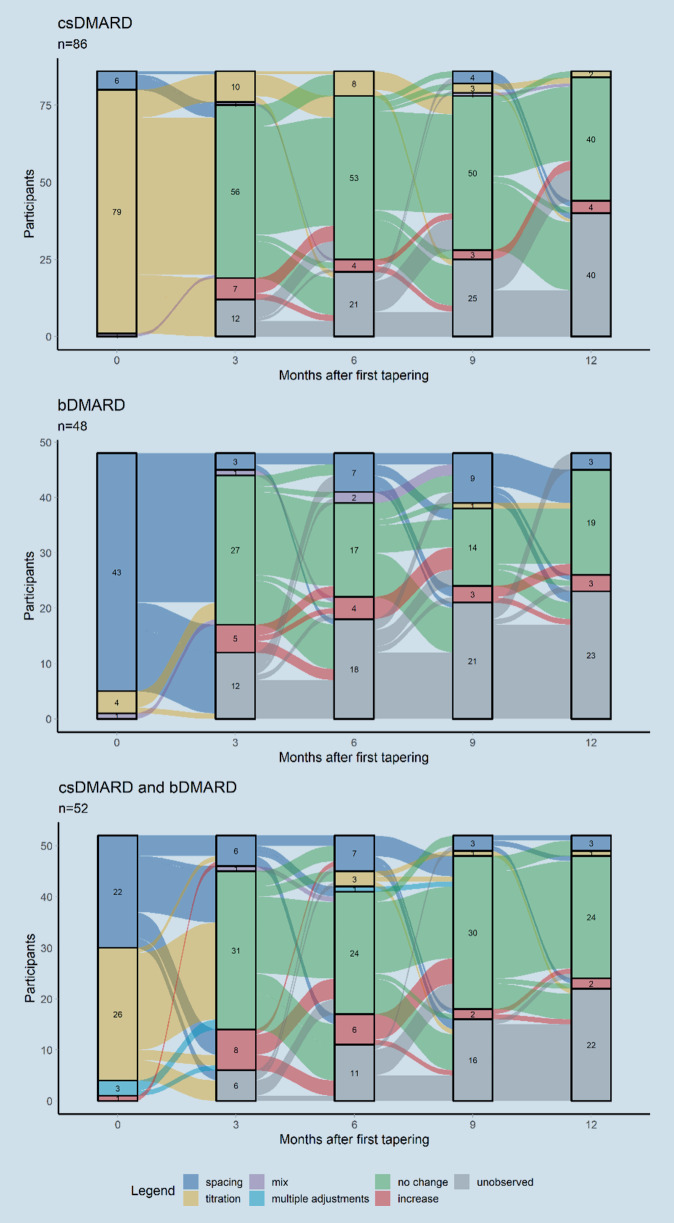
Alluvial plots depicting patient flows between treatment states for drug groups. *DMARDs* disease-modifying antirheumatic drugs, *bDMARDs* biological DMARDs, *csDMARDs* conventional synthetic DMARDs, *tsDMARDs* targeted synthetic DMARDs. Alluvials depict the transition to the state in the following 3 months; *spacing* increased interval with unchanged dosage, *titration* unchanged interval with decreased dosage, *mix* adjustment of both interval and dosage, *multiple adjustments* adjustment of more than one substance, *increase* increased dosage in at least one substance

Figure 2 depicts the patient flow between tapering strategies (titration, spacing, mix, multiple adjustments) as well as no change of DMARDs or increase of DMARDs or unobserved. Investigation of tapering patterns reveals that most patients were treated with one iteration of the tapering strategy throughout the observational period only. 25/52 (48.1%) patients in combination treatment initiated tapering with bDMARDs first, while 23/52 (44.2%) patients tapered csDMARDs first. 7/86 patients (8.1%) who were treated with csDMARDs only at baseline received re-increase of the dose of DMARDs at their first follow-up visit. Patients treated with bDMARDs received a re-increased dose in 10.4% (5/48 patients), while the highest number of re-increases was observed for patients treated with a combination of csDMARDs and bDMARDs, with 15.4% (8/52) at their first visit post-tapering. The majority of patients received an unchanged dosage at the first visit after tapering with 56/74 (75.7%) in csDMARD only, 27/36 (75.0%) in bDMARD only, and 31/46 (67.4%) in the csDMARD and bDMARD combination group.

Multiple iterations of titration or spacing were observed for 53/200 (26.5%) patients. Of these, 29/54 patients spaced their dosage at least twice (bDMARD: 17/18 patients; csDMARD and bDMARD: 12/15 patients). 18/20 patients titrated their dosage of csDMARDs twice, 2/20 patients received three iterations.

Increasing numbers of unobserved visits indicate that either the patients missed appointments or were not enrolled long enough to contribute more information. One reason for missed appointments could be the COVID-19 pandemic, which especially required reduction of contacts for patients with RA. Investigation of the subgroup of patients who were observable 12 months after the first tapering was confined to 101 patients. Of these, 46 received csDMARDs only, 25 received bDMARDs only, and 30 received combination treatment at the time of first tapering. It is apparent that a fraction of patients skipped appointments but were largely able to remain on an unchanged DMARD dosage later on. Most patients with failed tapering, i.e., increased dosage at one visit, were also able to remain on unchanged doses at the following visits.

### Tapering patterns by substance

**Fig. 3:**
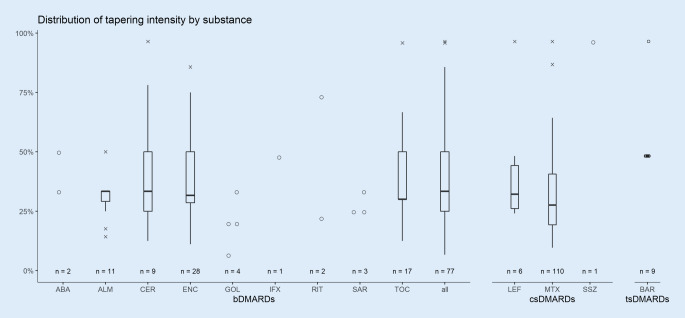
Comparison of tapering intensity measured as dose reduction in percent by substance. *DMARDs* disease-modifying antirheumatic drugs, *bDMARDs* biological DMARDs, *csDMARDs* conventional synthetic DMARDs, *tsDMARDs* targeted synthetic DMARDs, *ABA* abatacept, *ALM* adalimumab, *CER* certolizumab pegol, *ENC* etanercept, *GOL* golimumab, *IFX* infliximab, *RIT* rituximab, *SAR* sarilumab, *TOC* tocilizumab, *LEF* leflunomide, *MTX* methotrexate, *SSZ* sulfasalazine, *BAR* baricitinib

Investigation of tapering by substance groups shows heterogeneity of tapering approaches between substances (Fig. 3). The most frequently tapered bDMARD was etanercept (ENC). Tapering of ENC was observed for 28/77 patients (36%), with median tapering intensity of 32% compared to the dosage applied at the previous visit and substantial variation ranging from modest reductions of only 11% to a maximum of 86%. However, the IQR of 21 percentage points suggests that most patients were tapered to between 29% and 50% in comparison to the dosage at the previous visit. Tocilizumab (TOC) was tapered in 17/77 (22%) patients with a similar distribution (IQR: 20 percentage points). A more conservative approach was observed for patients treated with adalimumab (ADA). Here, 11/77 patients (14%) received tapering with a low variation in dose reduction of median 33% and an IQR of only 2 percentage points. Other bDMARDs were applied for smaller fractions of the population only (Fig. 3). Discontinuation of bDMARDs was not observed.

csDMARD tapering mostly focused on methotrexate (MTX), with 110/117 patients (94%) in this substance group. Tapering of leflunomide (LEF) or sulfasalazine (SSZ) was observed in six patients (5%) and one patient (1%), respectively. The median tapering intensity of MTX was 29% compared to the previous visit, with one quarter of patients being tapered by 42% or more. The minimal observed dose reduction of MTX was 10%. Eleven patients discontinued MTX following decreased or at least constant disease activity in comparison to the previous visit.

Tapering of tsDMARDs was observed for 9 patients who were all treated with baricitinib (BAR) only. All patients but one were tapered by 50% compared to the previous visit.

Overall, only 5/200 (2.5%) patients received glucocorticoids after a flare. Two of the patients were treated with bDMARD monotherapy and three with csDMARD monotherapy. For one patient who was treated with glucocorticoids after a flare, the observed bDMARD dose was increased.

## Discussion

We present results on real-life DMARD tapering strategies in RA patients following SDM. Our results indicate that rheumatologists require highly flexible strategies to taper DMARDs in RA patients. With the German guideline for managing RA highlighting the need to explore tapering approaches with regard to titration or spacing and intensity of dose reductions [2], our results provide new insights into this question from daily practice.

Whereas csDMARDs were mostly titrated, i.e., tapering by dose reductions while keeping treatment intervals constant, the opposite was seen for bDMARDs, where spacing of injections was applied in 89.5% of patients. Every fourth patient in our sample was treated with multiple iterations of either titration or spacing. Close to one half of patients treated with a combination of csDMARDs and bDMARDs had tapering of bDMARDs initiated first (48.1%), while csDMARDs were first tapered in 44.2% of patients with combination treatment. Further, tapering intensity in terms of relative dose reductions compared to previous visits covered a wide range, from only modest adjustments up to single cases of withdrawal. Time until first tapering was also highly individual, with median time to first tapering ranging from 108 days for csDMARDs up to 189 days for bDMARDs. These findings highlight the apparent need for individualization, as a persistent remission of at least 6 months was a prerequisite for enrolment and would have allowed an immediate start of tapering of DMARDs in all participants.

The distribution of relative dose reductions indicates heterogeneity of the applied tapering for both cs- and bDMARDs, with median dose reductions of 25% and 30%, respectively (IQR: 30% and 28%, respectively). Interestingly, most patients were tapered only once throughout the 12 months following the first tapering. Together with the overall low number of dose re-increases following tapering, this might indicate a more conservative tapering approach by rheumatologists in clinical practice compared to clinical trials, where spacing or titration of 50% is frequently required by study protocols [4, 6, 7].

Whether DMARDs are tapered by titration or spacing is related to the available dosage of substances. For example, MTX is available in several doses ranging from 7.5 mg up to 50 mg per injection in Germany, although 25 mg is recommended as the maximum dose per injection in the German guideline [2]. In contrast, ENC, the most applied bDMARD in our sample, is only available in two different concentrations of 25 mg or 50 mg for subcutaneous injection. Substances which are also available for intravenous injection could be tapered more individually; however, intravenous injection is rarely used in daily practice. We hypothesize that an increased diversity of available doses could enhance the ability of rheumatologists to further individualize tapering of bDMARDs to patients’ needs. With patients being reluctant to frequent changes of their therapy [12], which seems to contradict itself (since the application of a distance strategy would change the interval by definition): From the point of view of the practitioners, it could therefore be more advantageous to maintain the interval to which the patient is already accustomed.

Evidence on tapering DMARDs in daily clinical practice is scarce [13]. Observation of patients treated with MTX and concomitant adalimumab revealed—similar to our results—heterogeneity in the applied tapering strategies [14]. A tapered MTX dosage was observed in 26.7% of patients after 52 weeks, which increased to 47% after 104 weeks. Furthermore, 20% of patients also discontinued adalimumab due to ongoing remission. Existing evidence on treatment patterns in RA often focusses on persistence or discontinuation of DMARDs [15–17]. Persistence with tofacitinib in RA was estimated at 62.7% after 1 year, with only 12% of patients stating the reason why treatment was stopped [15]. Harnett et al. [16] found that 28% of patients receiving treatment with tumor necrosis factor inhibitors (TNFi) and concomitant csDMARDs were no longer taking csDMARDs at the time of their last prescription. However, reasons for discontinuation were not explored and may be based on inefficacy, poor tolerance, or indeed sustained remission. Hence, persistence data on single agents do not necessarily reflect successful tapering and stopping of DMARDs in sustained-remission patients. Our analysis extends the description of observed tapering strategies across substance groups beyond persistence rates or binary indicators of current treatment. Although we do not identify clear-cut treatment patterns in our data, our findings highlight the required degree of individualization of treatment approaches when tapering DMARDs in RA.

Several important hopes and concerns of patients and rheumatologists with regard to pharmaceutical treatment have been identified in qualitative research [18–20]. A narrative review identified a great need for more evidence on tapering in clinical practice as well as patient perspectives when tapering bDMARDs [18]. Chan et al. [19] investigated patient perspectives on bDMARD tapering and formulated overarching themes based on focus group interviews with 43 participants. The themes indicated that continuous therapy with stable disease activity and quality of life was preferred, but the perspective of taking less medication is welcome. Most recent research by Hazlewood et al. is based on individual interviews and focus groups of 28 patients and 23 rheumatologists [20]. Results suggested that both parties inherit diverse attitudes towards tapering of DMARDs. By providing details of applied tapering strategies across substance groups and variation of tapering intensity across substances, our findings highlight the requirement for individualized tapering approaches. Additionally, the authors find that both rheumatologists and patients report the concern of flares and loss of disease control as one of the main issues related to tapering [20]. This concern was also highlighted in early 2020 with the first wave of the COVID-19 pandemic in Germany by the German Association for Rheumatology (DGRh) [21]. However, we did not identify differences in the risk of flares or loss of remission, disease activity, or quality of life when comparing the study population of patients with tapering compared to continuation of treatment [22].

Finally, individualized tapering of DMARDs should be investigated from the health economic perspective. Available evidence suggests significant potential for cost savings from the payers’ and societal perspective, but heavily relies on evidence from clinical research [23–27]. Extending the scope of health economic evaluations to individualized tapering strategies, in particular the more conservative approach with less dose reduction, could help decision makers to better assess the potential of tapering DMARDs in RA under real-world circumstances.

## Limitations

Our analyses have several limitations. First, patients were selected by rheumatologists and thus might represent only a subpopulation of RA patients with positive attitudes toward tapering in general. The presented results, however, highlight the heterogeneity in applied tapering strategies based on SDM in daily clinical practice and offer useful insights for patients, rheumatologists, and decision makers aiming to improve the management of RA. Second, the distribution of patients across practices is not uniform and reveals that one large practice with seven physicians contributed close to one third of patients. The comparison of patient characteristics between the top two recruiting rheumatology practices, accounting for 47% of the sample, however, revealed no differences in comparison to the remaining study population. Third, due to the continuous recruitment until September 2020 and recommendations to avoid tapering after the start of the COVID-19 pandemic, the number of observable patients decreased during follow-up until March 2021. We chose to present the flow of patients across treatment states including unobserved periods. Patients with a loss in follow-up due to participation consent withdrawal were not observable due to data protection regulations. We addressed this by investigating treatment patterns for the subgroup of patients who were observable for 12 months after first tapering and did not find differences in the observed patient flows across substance groups.

## Conclusion

Tapering DMARDs based on SDM allows rheumatologists and patients to develop feasible paths to decrease the drug burden in RA. We have added substantial evidence to the question of how tapering based on SDM is applied by rheumatologists in daily clinical practice. Our findings show that individualized tapering based on SDM is possible even in complex situations, such as the COVID-19 pandemic. No meaningful worsening in disease activity and quality of life could be observed after tapering. The found heterogeneity of tapering approaches across substance groups emphasizes the need for further research in this regard. Identification and recognition of individualized approaches to tapering will help to further improve the management of RA for both patients and rheumatologists.

### Supplementary Information

Tab. S1 Classification of substance group based on available antirheumatic substances in Germany
Tab. S2 Descriptive statistics of the study population stratified by the top 2 recruiting rheumatology practices and remaining 16 rheumatology practices
Tab. S3 Descriptive statistics of the study population at 6 months after first tapering
Tab. S4 Descriptive statistics of the study population at 12 months after first tapering
Fig. S1 Time until first tapering (in days) after enrollment by substance group
Fig. S2 Alluvial plots depicting patient flows between treatment states for drug groups for patients observable at baseline and 12 months after first tapering

## Declarations

All studies mentioned here,were performed in compliance with the principles of the declaration of Helsinki and Good Clinical Practice (GCP).
