# Supplementary material for: Treatment patterns of individualized real-life tapering approaches based on shared decision-making in rheumatoid arthritis
Source: Z Rheumatol. 2023 Jun 23;83(2):142–50. doi: 10.1007/s00393-023-01380-z (PMC10901995; doi:10.1007/s00393-023-01380-z)
Supplement: Supplementary file 1 — Tab. S1 Classification of substance group based on available antirheumatic substances in Germany Tab. S2 Descriptive statistics of the study population stratified by the top 2 recruiting rheumatology practices and remaining 16 rheumatology practices Tab. S3 Descriptive statistics of the study population at 6 months after first tapering Tab. S4 Descriptive statistics of the study population at 12 months after first tapering Fig. S1 Time until first tapering (in days) after enrollment by substance group Fig. S2 Alluvial plots depicting patient flows between treatment states for drug groups for patients observable at baseline and 12 months after first tapering [file 393_2023_1380_MOESM1_ESM.pdf]

Supplementary Material

Table S1 Classification of substance group based on available anti-rheumatic substances in Germany

Substance Group	Abbreviation	Name
bDMARD	ABA[1]	Abatacept
bDMARD	AK	Anakinra
bDMARD	ALM[1]	Adalimumab
bDMARD	BEL	Belimumab
bDMARD	CAN	Canakinumab
bDMARD	CER[1]	Certolizumab Pegol
bDMARD	ENC[1]	Etanercept
bDMARD	GOL[1]	Golimumab
bDMARD	GUS	Guselkumab
bDMARD	IFX[1]	Infliximab
bDMARD	IXE	Ixekizumab
bDMARD	RIT[1]	Rituximab
bDMARD	SAR[1]	Sarilumab
bDMARD	SEC	Secukinumab
bDMARD	SIR	Sirukumab
bDMARD	TOC[1]	Tocilizumab
bDMARD	UST	Ustekinumab
csDMARD	LEF[1]	Leflunomid
csDMARD	MTX[1]	Methotrexat
csDMARD	SSZ[1]	Sulfasalazin
tsDMARD	APR	Apremilast
tsDMARD	BAR[1]	Baricitinib
tsDMARD	TOF	Tofacitinib
tsDMARD	UPA	Upadacitinib

[1]Observed prescriptions of substances in study sample

Table S2 Descriptive statistics study population stratified by top 2 recruiting rheumatologist practices and remaining 16 rheumatologist practices

	Top 2 Rheumatologist Practices n = 94		Remaining 16 Rheumatologist Practices n = 106		
	mean (n)	sd (%)	mean (n)	sd (%)	
Age	58.39	13.38	56.55	15.52	
Female	0.79	0.41	0.73	0.44	
RA duration	7.92	6.73	10.51	9.96	**
DAS28-ESR	1.95	0.67	2.04	0.67	
DAS28-CRP	1.73	0.43	1.86	0.54	*
CDAI	2.22	2.23	2.49	2.67	
Remission overall	0.97	0.18	0.91	0.29	
Remission DAS28-ESR	0.81	0.39	0.84	0.37	
Remission DAS28-CRP	0.96	0.21	0.84	0.37	**
Remission CDAI	0.70	0.46	0.62	0.49	
EQ5D	0.92	0.10	0.93	0.10	
RADAI	1.27	1.15	1.28	1.23	

Note: Descriptive statistics study population stratified by top 2 recruiting rheumatologist practices and remaining 16 rheumatologist practices.
[1]standard deviation, *** p<0.01, ** p < 0.05, * p< 0.1

Table S3 Descriptive statistics of study population at 6 months after first tapering

	csDMARD[1] n = 65		bDMARD[2] n = 30		csDMARD and bDMARD n = 41		tsDMARD[3] n = 4		csDMARD and tsDMARD n = 3		overall n = 143	
	mean (n)	sd[4] (%)	mean (n)	sd[4] (%)	mean (n)	sd[4] (%)	mean (n)	sd[4] (%)	mean (n)	sd[4] (%)	mean (n)	sd[4] (%)
Age	62.2	12.5	53.6	17.3	54.5	11.9	66.3	5.1	52.3	12.9	58.1	13.9
Female	51	78.5	20	69.0	30	73.2	3	75.0	3	100.0	107	75.4
RA duration	6.1	6.3	12.9	10.4	12.22	11.6	10.23	5.6	3.63	2.2	9.34	9.5
DAS28	2.22	0.67	1.94	0.75	2.17	1.03	2.78	0.51	1.55	0.49	2.16	0.81
DAS28-CRP	1.90	0.48	1.71	0.49	2.01	0.73	1.80	0.70	2.10	0.69	1.89	0.58
CDAI	2.67	2.43	2.49	2.30	3.47	4.15	3.38	4.46	3.27	1.94	2.90	3.05
remission	56	88.9	28	93.3	34	85.0	3	75.0	2	66.7	123	87.9
seropositive	14	38.9	15	65.2	16	69.6	2	100.0	1	50.0	48	55.8
EQ5D	0.89	0.17	0.92	0.15	0.90	0.13	0.92	0.10	0.92	0.06	0.90	0.15
RADAI	1.41	1.52	1.47	1.41	1.66	1.74	1.40	1.14	2.07	1.55	1.51	1.54

Note: Descriptive statistics of study population. [1]conventional-synthetic disease-modifying anti-rheumatic drug (DMARD), [2]biological DMARD, [3]targeted-synthetic DMARD, [4]standard deviation

Table S4 Descriptive statistics of study population at 12 months after first tapering

	csDMARD[1] n = 46		bDMARD[2] n = 25		csDMARD and bDMARD n = 30		tsDMARD[3] n = 3		csDMARD and tsDMARD n = 3		overall n = 107	
	mean (n)	sd[4] (%)	mean (n)	sd[4] (%)	mean (n)	sd[4] (%)	mean (n)	sd[4] (%)	mean (n)	sd[4] (%)	mean (n)	sd[4] (%)
Age	61.3	11.1	51.4	17.7	53.2	10.8	64.0	7.2	52.7	13.1	56.5	13.4
Female	34	73.9	19	79.2	22	73.3	2	66.7	3	100.0	80	75.5
RA duration	6.1	5.3	12.3	10.4	12.79	11.5	8.95	5.8	2.47	0.2	9.42	9.2
DAS28	2.24	1.00	2.30	1.09	2.20	1.03	2.60	0.42	1.50	0.00	2.24	1.00
DAS28-CRP	2.15	0.88	1.98	0.76	2.11	0.77	1.33	0.06	2.03	0.61	2.06	0.80
CDAI	4.00	5.28	4.90	4.73	4.30	4.11	1.37	1.52	4.65	6.58	4.23	4.74
remission	34	73.9	20	80.0	25	83.3	3	100.0	2	66.7	84	78.5
seropositive	12	44.4	14	73.7	14	82.4	1	50.0	2	66.7	43	63.2
EQ5D	0.89	0.15	0.88	0.14	0.87	0.19	0.92	0.06	0.89	0.11	0.88	0.16
RADAI	1.52	1.58	2.01	1.44	1.76	1.47	1.13	1.03	2.14	1.78	1.71	1.50

Note: Descriptive statistics of study population. [1]conventional-synthetic disease-modifying anti-rheumatic drug (DMARD), [2]biological DMARD, [3]targeted-synthetic DMARD, [4]standard deviation

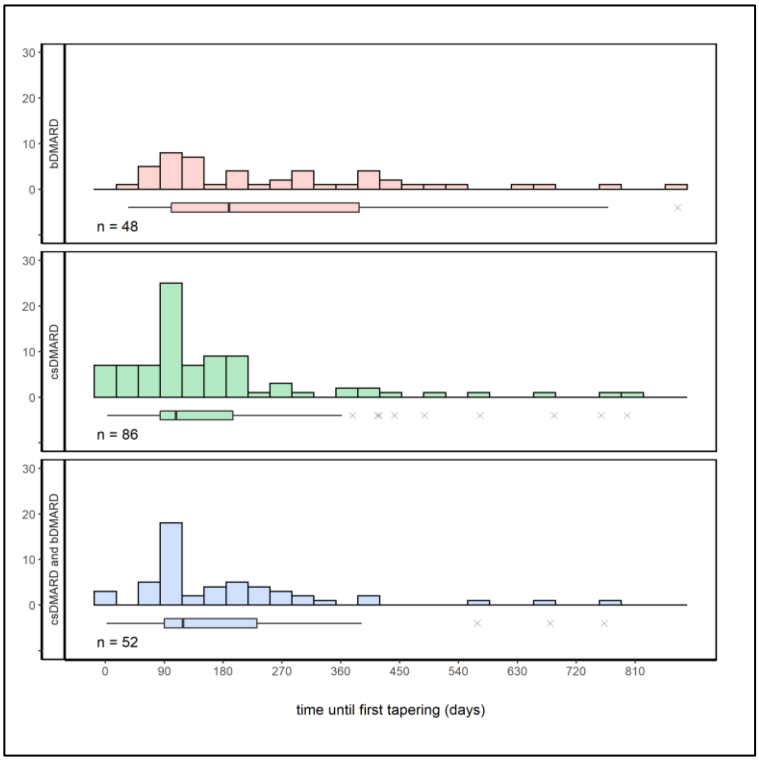

Figure S1 Time until first tapering in days after enrollment by substance group

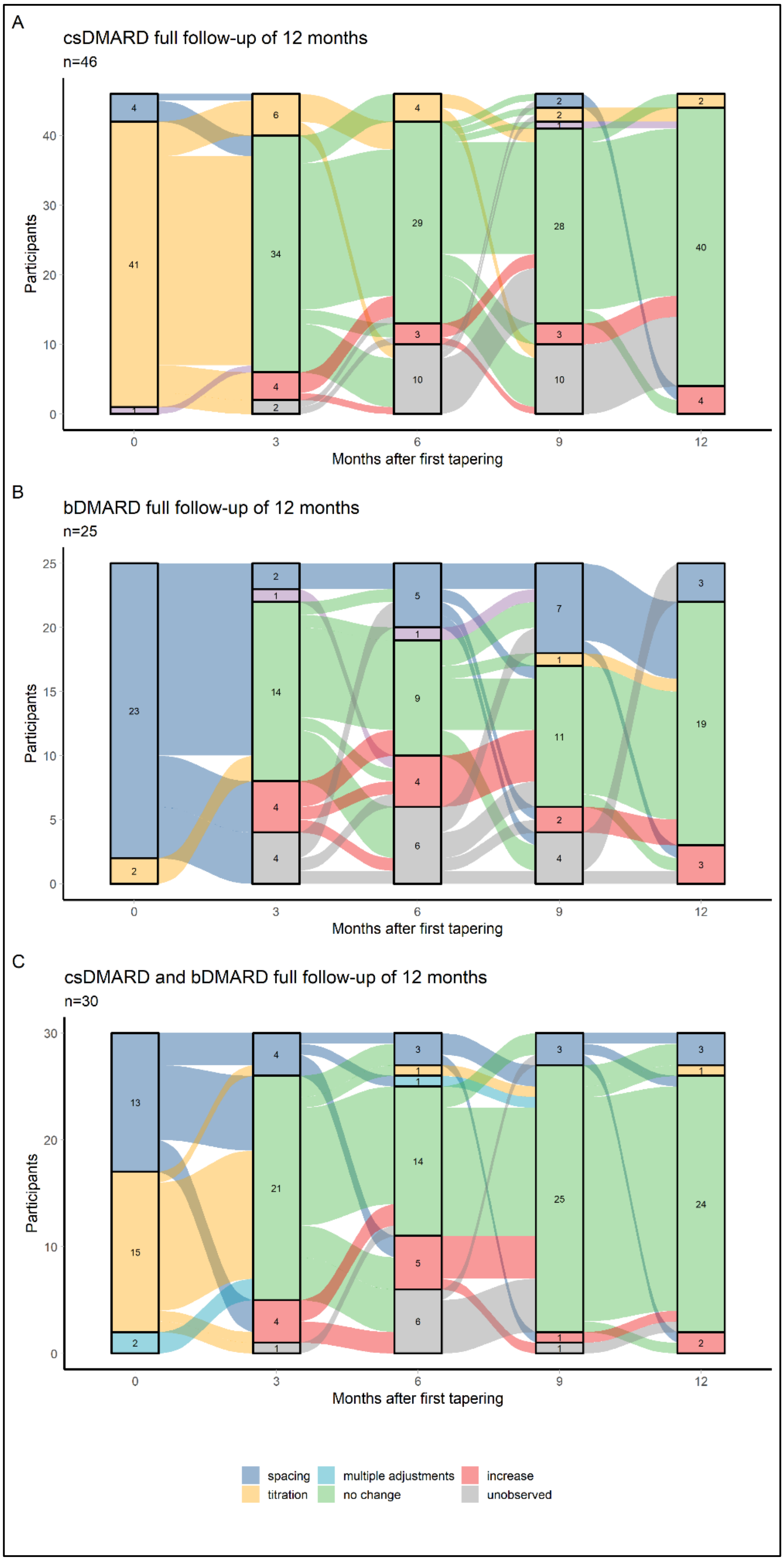

Figure S2 Alluvial plots depicting patient flows between treatment states for drug groups for patients observable at baseline and 12 months after first tapering
